# Risk factors for migration of retrievable covered expandable metallic stent in patients with persistent benign ureter strictures

**DOI:** 10.1007/s00345-024-04986-3

**Published:** 2024-04-30

**Authors:** Yuyu Xu, Xiezhao Li, Zhiduan Cai, Shuangxing Chen, Rui Zhu, Haishan Zhuang, ShawPong Wan, Guibin Xu

**Affiliations:** 1 Department of Urology, Key Laboratory of Biological Targeting Diagnosis, Therapy and Rehabilitation of Guangdong Higher Education Institutes, The Fifth Affiliated Hospital of Guangzhou Medical University, Guangzhou Medical University, Guangzhou, 510700 China; 2 Guangdong Provincial Key Laboratory of Urology, The First Affiliated Hospital of Guangzhou Medical University, Guangzhou Medical University, Guangzhou, 510230 China; 3 First People’s Hospital of Xiaoshan, Hangzhou, 311200 Zhejiang China

**Keywords:** Ureter stricture, RCEMS, Risk factor

## Abstract

**Purpose:**

The purpose of this study is to evaluate the incidence, risk factors, and salvage management of retrievable covered expandable metallic stent (RCEMS) migration in patients with persistent benign ureter strictures.

**Materials and methods:**

A retrospective study was performed on 117 consecutive patients who underwent implantation of RCEMS. Univariate and multivariate analyses were used to identify prognostic factors for stent migration, including stricture location and length, hydronephrosis–cortex ratio, ureteral dilation, and the diameter of the narrowest portion of the stricture.

**Results:**

Stent migration occurred in 22 (19.5%) of 113 patients who met inclusion criteria. Of the 22 patients, 16 (72.7%) had ordinary ureteral stricture, 3 (13.6%) had stricture in transplanted kidneys, and 3 patients (13.6%) had ureter stricture in orthotopic neobladders. The mean creatinine for the entire cohorts showed significant improvement (*p* = 0.038). Multivariate analysis identified the following prognostic factors for migration: distal ureteral stricture (*p* = 0.006), patients who underwent balloon dilation (*p* = 0.003), hydronephrosis–cortex ratio ≧10 (*p* = 0.017), larger diameter of wasting of RCEMS (*p* < 0.001), and patients with a shorter stricture length (*p* = 0.006). Salvage management was required in 4 of the 22 patients. The strictures in the remaining 18 patients improved with observation.

**Conclusions:**

Stent migration is more likely to occur in patients with the five prognostic factors mentioned above. Our study developed a nomogram to predict stent migration in patients with ureteral strictures treated using RCEMS.

## Introduction

Benign ureter stricture is often an iatrogenic complication of surgery for upper urinary tract disease and nephrolithiasis. It can compromise quality of life and impair renal function. Simple and short strictures may be amendable to dilation and/or internal ureterotomy. Strictures that failed simple management, hereafter referred to as persistent strictures, require reconstructive surgery. Such surgery can be complicated and at times have unsatisfactory results. Retrievable covered expandable metallic stents (RCEMS) have become a therapeutic option for persistent strictures [1, 2]. RCEMS can improve urine drainage and preserve renal function. However, adverse events related to RCEMS, such as stent migration, stent-induced mucosal hyperplasia, and stent obstruction, can diminish its success.

There are multiple factors that can influence RCEMS migration. They include the stricture’s location and length, the hydronephrosis–cortex ratio, the need for balloon dilation, and the diameter of wasting of RCEMS. The purpose of this study is to retrospectively evaluate the incidence, prognostic factors, and salvage management of stent migration. The study protocol was approved by our Institutional Ethics Committee.

## Material and methods

### Material

We retrospectively reviewed the clinical data of RCEMS placement in 113 patients in our center between January 2019 and December 2022. Written informed consent of the procedure was obtained from each patient before RCEMS insertion in accordance with the Helsinki Declaration. Inclusion criteria were patients with benign ureter strictures that failed conservative treatments such as dilation and/or internal ureterotomy and required further intervention. Patients who were lost after RCEMS placement were excluded from the analysis.

### Method

#### RCEMS insertion

Each patient underwent a contrasted CT evaluation of the stricture prior to RCEMS placement. RCEMS was inserted under fluoroscopic guidance. The RCEMS (Allium Medical Solutions Ltd., Israel) is made of two components: the stent and a 10 Fr. insertion system. A retrograde approach was chosen for all the cases. The procedure was performed under either general or regional anesthesia as per patient’s preference or anesthesiologist’s recommendation. After identifying the location and length of the stenotic segment with retrograde pyelography, a 0.032″ guidewire was introduced, passing through the stenotic segment up to the renal pelvis. An RCEMS with a circumference of 30 Fr. and a length of 10 cm within its 10 Fr insertion system was introduced over the guidewire and advanced to desirable position, where the RCEMS was located at equal distances from both ends of the stenotic segment. If the insertion system met resistance in passing through the stenotic segment, the stenotic segment was dilated using a balloon catheter (15F/10 cm, Bard, U. S. A.), until the ‘wasting’ sign disappeared under fluoroscopic monitoring. Once the RCEMS was in position, the trigger on the deployment device was pulled to release the stent. The stent would self-expand into a cylindrical shape, matching the anatomy of the normal ureter and exerting an outward force on the ureteral wall.

#### Follow-up and diagnosis of RCEMS migration

**Fig. 1:**
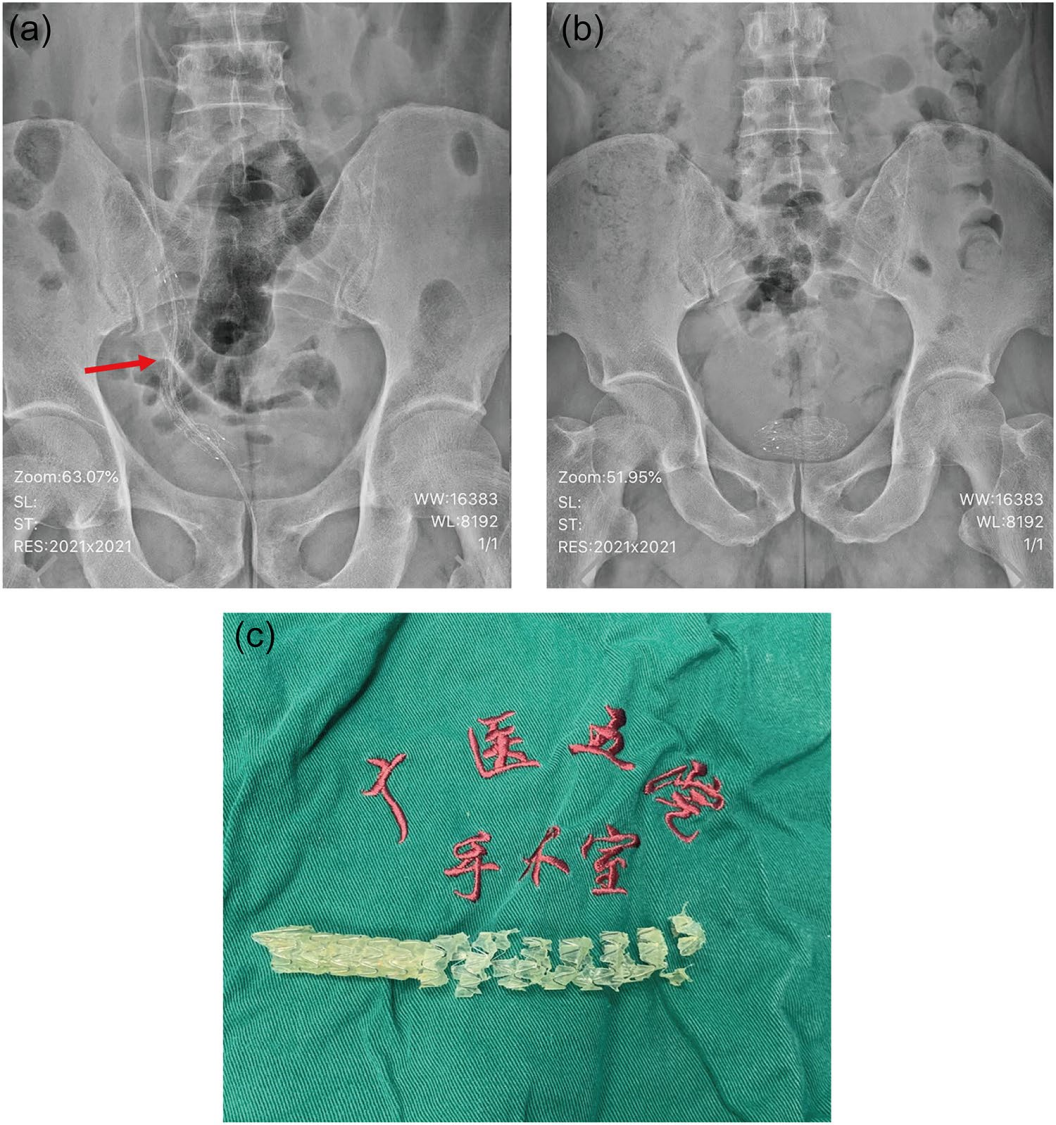
**a** KUB on the first day after RCEMS placement. **b** Stent migrated into the bladder. **c** Retrieved RCEMS after migration

Serum creatinine was obtained pre- and post-operatively and serially thereafter as needed for the assessment of renal function. Hydronephrosis was evaluated using ultrasonography. We calculated the ratio between the largest transverse length of the renal pelvis to the largest transverse length of the renal cortex on the same CT image as the hydronephrosis–cortex ratio. Although most studies describe hydronephrosis of renal pelvis using antero-posterior diameter measurements, we believe the hydronephrosis–cortex ratio can be a better indicator to describe the hydronephrosis. KUB was obtained on the first day after surgery, then followed up at 1, 3, and every 6 months thereafter. When patients with RCEMS placement experienced renal colic or fever, an ultrasonographic examination, KUB, or both were performed. Stent migration was defined as the relocation of a placed stent to above or below the stricture (Fig. 1a, b and c). If stent migration was evident, ureteroscopy was performed to confirm the degree of improvement of the ureteral stenosis. If the stent migrated to the distal end of the stenosis, it was removed retrogradely; if the stent migrated up to the upper end of the stenosis, it was retrieved retrogradely if feasible; otherwise, it was removed by percutaneous approach. The improvement of stenosis was assessed by either retrograde pyelography or by ureteroscopy. If stenosis showed no improvement, salvage treatment including placement of double-J stent, insertion of another RCEMS, or placement of a nephrostomy tube was performed.

### Statistical analyses

Related data are expressed either as numbers and percentages of the patients, or as ranges and variables. The chi-squared or Fisher’s exact test was used to compare the categorical variables. A *P* < 0.05 was considered statistically significant. Univariate and multivariate analyses to identify prognostic factors for RCEMS migration were performed using a proportional hazards model proposed by Fine and Gray. Renal cortical thickness is defined as the thickness of the maximum cross-section of the three layers of kidney. Hydronephrosis–cortex ratio is defined as the maximum diameter of hydronephrosis divided by renal cortical thickness. We included age, gender, necessity for balloon dilation, stricture location (proximal vs middle vs distal), stricture length (≦10 mm *vs* > 10 and < 20 mm *vs* ≧20 mm), hydronephrosis–cortex ratio (≦5 *vs* > 5 and < 10 *vs* ≧10), and diameter of wasting of RCEMS into the model. Odd ratios (OR) with 95% confidence intervals (CIs) were calculated. Factors with *P* < 0.05 by univariate analysis and highly correlated factors with *P* > 0.05 were further analyzed in a multivariate analysis. All analyses were performed using SPSS software, version 14.0.

## Results

A total of 117 patients with persistent benign ureter stricture underwent successful 30Fr., 10-cm RCEMS implantation. Four patients were lost to follow-up and were excluded. 113 patients entered the final analysis. Patients, stricture characteristics, and ancillary treatments in this study are shown in Table 1. Nine patients experienced fever on the first post-operative day; 15 patients had stent related urinary tract infection; and seven patients developed stent encrustation.

**Table 1:** Patients demographics and perioperative characteristics

Variables	Value
Age, median (range)	45 (11–84)
Gender	
Male	64 (56.64%)
Female	49 (43.36%)
Location, n (%)	
Proximal	24 (21.24%)
Middle	38 (33.63%)
Distal	51 (45.13%)
Stricture etiology, n (%)	
Persistent strictures after prior treatment	97 (85.84%)
Transplant ureteral stricture	8 (7.08%)
Ureteroileal anastomosis strictures	5 (4.42%)
Others	3 (2.66%)
Prior treatments, n	
Double-J stent	113
Dilation	69
Reconstructive surgery	65
Incision–dilation	9
Percutaneous nephrostomy	17
Grade of stricture length (mm), n (%)	
≦ 10	31 (27.43%)
10–20	40 (36.40%)
≧ 20	42 (36.17%)
Balloon dilation or not, n (%)	
Yes	75 (66.37%)
No	38 (33.63%)
Diameter of wasting of RCEMS one day after surgery, mean ± standard deviation	5.74 ± 1.55 mm
Patients with RCEMS migration, n (%)	22 (19.5%)
Observation without further treatment	18 (15.9%)
Ancillary treatments, n (%)	4 (3.5%)
Double-J stent	2 (1.76%)
Percutaneous nephrostomy	1 (0.88%)
Open surgery for ureteroplasty	1 (0.88%)

Creatinine values of the entire cohorts (µmol/L), mean ± standard deviation		
Pre-operative	90.00 ± 56.00	*P* = 0.038
Post-operative	88.90 ± 51.20	
Creatinine values of RCEMS migration cohorts (µmol/L), mean ± standard deviation		
Pre-operative	83.85 ± 55.38	*P* = 0.270
Post-operative	87.20 ± 52.72	
Creatinine values of RCEMS non-migration cohorts (µmol/L), mean ± standard deviation		
Pre-operative	91.80 ± 56.00	*P* = 0.079
Post-operative	90.90 ± 52.00	

### Incidences of stent migration

Stent migration was observed in 22 patients (19.5%) with a median of 15 months (range, 0.5–40) from the stent placement. Of these 22 patients, migration occurred in 16 (72.7%), 3 (13.6%), and 3 (13.6%) patients with ordinary, transplanted ureter, and orthotopic neobladder ureteral strictures, respectively.

### Renal function

The mean creatinine for the entire cohorts showed significant decrease from 90 µmol/L to 88.9 µmol/L, *P* = 0.038. The creatinine level did not reveal any significant change (*P* = 0.27) in the 22 patients who experienced stent migration on sub-analysis. However, patients whose RCEMS migrated but required no intervention, the creatinine exhibited significant improvement. Whereas there was deterioration in the serum creatinine in the four patients whose migration of RCEMS required intervention; but due to the small number of the patients, the difference was not statistically significant.

### Salvage management

Four patients with stent migration who showed no improvement of the stricture underwent salvage management. Two patients were treated with double-J stent; one underwent percutaneous nephrostomy tube placement, and one underwent open ureteroplasty. Eighteeen patients had the stents removed without the need for further treatment. On follow-up, these 18 patients did not experience any clinical symptoms and ultrasonography showed no increased hydronephrosis.

### Risk factors for RCEMS migration

**Fig. 2:**
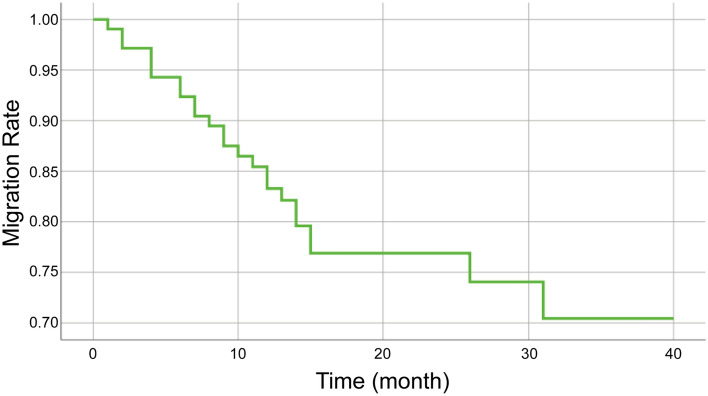
Kaplan–Meier curve for estimation of the RCEMS migration rate

Incidences of stent migration stratified to various factors are shown in Table 2; a Kaplan–Meier curve for estimation of the migration rate is shown in Fig. 2. The incidence of stent migration was significantly higher in patients who required balloon dilation and in patients with distal ureteral stricture.

**Table 2:** Univariate analysis and multivariate analysis of RCEMS migration

Variable	Univariate analysis			Multivariate analysis		
	OR	95% CI	P	OR	95% CI	P
Age	0.983	0.953–1.014	0.289			
Gender	1.405	0.569–3.472	0.461			
Location			0.367			0.071
Proximal	1			1		
Middle	0.933	0.256–3.399	0.917	0.746	0.141–3.934	0.729
Distal	1.901	0.696–5.193	0.210	4.761	1.046–21.678	0.044
Grade of stricture length			0.023			0.006
≦10 mm	1		0.028	1		
> 10 and < 20 mm	0.336	0.113–0.999	0.05	0.128	0.026–0.623	0.011
≧20 mm	0.214	0.066–0.697	0.01	0.085	0.017–0.426	0.003
Hydronephrosis–cortex ratio			0.377			0.059
≦5	1			1		
> 5 and < 10	1.173	0.225–6.105	0.850	1.083	0.135–8.691	0.940
≧10	3.079	0.635–14.929	0.163	13.396	1.584–113.275	0.017
Balloon dilation	3.025	1.198–7.641	0.019	7.879	1.995–31.111	0.003
Diameter of wasting of RCEMS one day after surgery (mm)	8.726	2.952–25.794	< 0.001	2.417	1.554–3.760	< 0.001

## Discussion

The management of ureteral obstruction is a challenging task. Open surgery is often used in ureteral reconstruction. However, reconstructive surgery is not always feasible, especially in high-risk patients, i.e., ASA ≧3. An endourological approach has become the first-line treatment due to its minimally invasive nature. Various surgical techniques such as ureteral dilation, double-J stenting, and percutaneous nephrostomy have been described. However, these techniques carry considerable failure rates and have negative impacts on quality of life.

The use of longer-term ureteral stenting for ureteral stricture treatment has increased over the years. Metal stents have been shown to yield better outcomes and longer stent patency than short-term non-metallic stents for benign or malignant ureteral obstruction [3–5]. There are adverse events associated with metal ureteral stents. These include but are not limited to infection, lower urinary tract symptoms, mucosa ingrowth, stent migration, encrustations, and difficulties in removing the implanted stents [6, 7]. To overcome the condition of mucosa ingrowth and difficulties in removing the stent, several covered stents with coating technology for anti-mucosa hyperplasia have been developed. Covered stents may increase the prospect of stent migration. Stent migration has been reported to range from 12.5% to 43% [3, 8–10]. In our study, the rate of migration is 20.2%. In eight patients, the stents migrated upward into the renal pelvis; in 14 patients, the stents migrated downward within the ureter or into the bladder. Stent migration is often related to the location of the stricture.

Factors contributing to migration were not well defined prior to this study. Many conditions contribute to the risks of stent migration. These include but are not limited to the nature of ureteral stricture, stricture location and length, hydronephrosis–cortex ratio, and whether balloon dilation was required. Hence, we performed a multivariate prognostic analysis to predict the stent migration in patients treated with RCEMS. Our retrospective analysis revealed five significant risk factors for stent migration: having a distal ureteral stricture (OR = 4.761; *p* = 0.044), undergoing balloon dilation (OR = 7.879; *p* = 0.003), hydronephrosis–cortex ratio ≧10 (OR = 13.396; *p* = 0.017), larger diameter of wasting of RCEMS (OR = 2.417; *p* < 0.001), and having a shorter stricture length (*p* = 0.006). We posit that the greater the hydronephrosis–cortex ratio, the worse the renal urinary function; hence, the deterioration of the natural smooth muscle tone of the collection system. The larger diameter of wasting of RCEMS and shorter stricture length will result in the reduction of the anchoring ability of the RCEMS.

Renal scintigraphy (99mTc-DPTA clearance) is the best method to measure unilateral renal function and obstruction as well as for follow-up evaluation. Unfortunately, the renal scintigraphy is quite expensive in China and is not covered by the national health insurance. Consequently, few patients consented to the test. Alternatively, we used serial serum creatinine and ultrasonography for follow-up. We found that there was significant improvement in the renal function for the entire study cohorts after RCEMS placement (*P* = 0.038). On the other hand, there was no significant improvement in serum creatinine in the 22 patients who had RCEMS migration (*P* = 0.27). Therefore, serial creatinine level could be useful, though crude, for follow-up of renal function.

To prevent migration, stents with various anti-migration features, such as increased stent diameter, double layering, and stent fixation methods, were introduced. Stent migration may necessitate repeat interventions. The cost of RCEMS might also be an issue, especially when migration occurs within 6 months of the stent placement. In our study, there were four migrations that required repeat interventions, all due to a lack of stricture improvement. Eighteen migrated stents were removed but without the need for further intervention. In our experience, stent migration may be a manifestation of improvement in stenosis. Most patients in our series achieved satisfactory results even after stent migration. Moskovitz et al. reported that 42.9% (3/7) of patients had excellent outcomes despite stent migration and required no further intervention [8].

Transplant ureteral stricture (TUS) is a unique problem that occurs in about 5% of the patients [11]. Proper and timely treatment of a migrated stent can avoid injury to the transplanted kidney. RCEMS placement is recommended for TUS refractory to simple stenting [12]. Cao et al. reported an 87.5% successful rate and 25% migration rate for RCEMS implanted for TUS [13]. Subgroup analysis in our study revealed a 37.5% migration rate for TUS, which was higher than the overall migration rate. This study might provide some insights that help a treating physician to identify the optimal patients for RCEMS implantation and to predict the probability of stent migration in advance.

Ureteroileal anastomosis strictures are a serious complication after orthotopic urinary diversion. They have been reported to occur in 10%–16% of the patients [14]. Open revision remains the gold standard for the management of such strictures. However, endourological procedures such as balloon dilatation and endoureterotomy, either with cold knife or laser, are minimally invasive options associated with lower morbidity. Unfortunately, these techniques also yield lower success rates. RCEMS has been shown to be an attractive long-term and cost-effective solution for ureteroileal strictures [15], a potential alternative to open revision.

The limitations of this study included the broader inclusion criteria, namely the younger patients. These patients elected RECMS as the treatment of choice, even though they may be suitable for reconstructive surgery. Nevertheless, these young patients fared very well with RCEMS. Furthermore, prior RECMS placement should not adversely affect further reconstructive surgery. We had several patients lost in follow-up that could alter the final outcomes. We added a Kaplan–Meier curve to mitigate this potential discrepancy. Due to economical constraints, we did not use diuretic nuclear renal scan for follow-up obstruction, a key deficiency that could impact our conclusion. Finally, the retrospective nature and the relatively small patient population of this study have their own inherent limitations.

## Conclusion

Our research demonstrated that distal ureteral stricture (*p* = 0.006), undergoing balloon dilation (*p* = 0.003), hydronephrosis–cortex ratio ≧10 (*p* = 0.017), larger diameter of wasting of RCEMS (*p* < 0.001), and having a shorter stricture length (*p* = 0.006) are risk factors for RCEMS migration.

## Data Availability

The data that support the findings of this study are available from the corresponding author upon reasonable request.

## Abbreviations

RCEMS: Retrievable covered expandable metallic stent
CT: Computerized tomography
KUB: Plain film of the abdomen
OR: Odd ratios
Cis: Confidence intervals
TUS: Transplant ureteral stricture
